# Repetitive Transcranial Magnetic Stimulation for the Treatment of Depression in a Real-World Setting: Findings from a Cohort Study

**DOI:** 10.3390/brainsci14090949

**Published:** 2024-09-22

**Authors:** Tiziano Prodi, Gabriele Pezzullo, Kevin La Monica, Alberto Priori, Matteo Vismara, Bernardo Dell’Osso, Beatrice Benatti

**Affiliations:** 1 Department of Psychiatry, Department of Biomedical and Clinical Sciences “Luigi Sacco”, University of Milan, ASST Fatebenefratelli-Sacco, 20157 Milan, Italy; tiziano.prodi@unimi.it (T.P.);; 2 “Aldo Ravelli” Center for Nanotechnology and Neurostimulation, University of Milan, 20122 Milan, Italy; 3 Department of Health Sciences, San Paolo Hospital, University of Milan, 20142 Milan, Italy; 4 Department of Psychiatry and Behavioral Sciences, Stanford University, Stanford, CA 94305, USA

**Keywords:** transcranial magnetic stimulation (TMS), depressive disorder, bipolar disorder, cognition, anxiety

## Abstract

Background/Objectives: In the past two decades, significant advancements in neuromodulation techniques have occurred, such as transcranial magnetic stimulation (TMS) for treatment-resistant depression (TRD). According to the assumption that repeated stimulation within a condensed timeframe can yield sustained efficacy, an accelerated protocol may be more effective in reducing time to response. With those premises, this study aimed to evaluate a sample of TRD patients treated with standard repetitive TMS (rTMS) and accelerated rTMS (arTMS). Methods: Nine subjects were treated with standard rTMS and 19 with arTMS. Psychometric assessment was made at the baseline and one week, one month, and three months after the treatment. A linear mixed-effect regression was performed along with other appropriate statistical analyses. Results: A significant improvement over time was observed for both depressive and cognitive symptoms. Moreover, considering the reduction in the Montgomery–Asberg Depression Rating Scale scores, a better treatment response was observed in subjects treated with arTMS (*p* < 0.05). Conclusions: Our findings showed a significant difference between the two protocols in terms of clinical response. Although further studies are needed to confirm the superiority of arTMS, the better cost-effectiveness of this technique should be considered.

## 1. Introduction

### 1.1. Neuromodulation in Depressive Episodes

In the past two decades, significant advancements have occurred in neuromodulation techniques, leading to their increased utilization in psychiatry and other fields. For instance, transcranial magnetic stimulation (TMS) utilizes brief, high-intensity electric pulses via a coil placed on the scalp to generate a focused magnetic field. This field induces electric currents in underlying brain tissue, activating local and distant neurons [1]. Currently, one of the primary applications of TMS is treatment-resistant depression (TRD) treatment [2]. TRD is typically defined as a condition in which patients with major depressive disorder (MDD) fail to respond to at least two distinct and appropriate antidepressant treatments administered at adequate doses and for a sufficient duration [3]. More than 20% of depressive cases are unresponsive to pharmacological treatment and—even among those who initially achieved remission—a relapse rate of 43% within one year of treatment was observed [4]. By now, the gold standard for TRD remains to be electroconvulsive therapy (ECT), especially in the presence of life-threatening conditions, high suicidal risk, or depression with psychotic symptoms [5]. However, it remains challenging to access and carries a significant stigma [6]. Moreover, treatments such as ECT or magnetic seizure therapy are considered more invasive treatment protocols because they involve the administration of anesthetic agents and sedation [7]. Consequently, alternative therapeutic methods like TMS could serve as a viable option [6]; indeed, Mutz and colleagues suggested that protocols such as high-frequency left repetitive TMS (rTMS), low-frequency right rTMS, bilateral rTMS, and transcranial direct current stimulation (tDCS) should be preferred to others with more limited evidence of efficacy [7]. Regarding tDCS, a recent review of the literature highlighted that this kind of treatment is more effective for MDD in patients under SSRI treatment [8]. Moreover, several clinical trials investigated different protocols, leading to a reduction in generalizability [8]. Conversely, considering the case of TMS protocols, significant advances in understanding and using this new technology have occurred in the last decade, with systematic reviews and meta-analyses consistently supporting its efficacy, showing response rates ranging from 50% to 80% [9,10,11].

### 1.2. The Case of Transcranial Magnetic Stimulation

TMS treatment, in addition to its effectiveness, is well tolerated with only a few and generally acceptable side effects [12]. The effects of TMS—in terms of both physiological and treatment impact—are influenced by the stimulation parameters, which include the target location, frequency/pattern, intensity of pulses, number of pulses per session, and total sessions throughout treatment. The most common protocol for depression involves rTMS with a 10 Hz frequency, targeted at the left dorsolateral prefrontal cortex (DLPFC) [13]. Clinical trials also validate the antidepressant effectiveness of 1 Hz rTMS on the right DLPFC and sequential bilateral rTMS targeting both sides of the prefrontal cortex [14]. This approach is supported by findings of hypoactivation in the left DLPFC and hyperactivation in the right DLPFC in depressed patients. Consequently, high-frequency (10 Hz) and low-frequency (1 Hz) stimulation can have excitatory or inhibitory effects, respectively [14]. The antidepressant effects of rTMS are associated with increased levels of brain-derived neurotrophic factor (BDNF), altered neurotransmitter release and binding, and synaptic strengthening or weakening through processes like long-term potentiation (LTP) or long-term depression (LTD) [1]. Neuroimaging studies have also consistently observed an indirect inhibitory functional connectivity from the left DLPFC to the subgenual anterior cingulate cortex (sgACC), which is known to be involved in abnormal emotional processing, sadness, depression severity, and response to various antidepressant therapies [15]. During a standard treatment course, stimulation sessions are generally administered daily, five days per week, for 20–30 sessions over 4–6 weeks [16]. Improvements in depressive symptoms are generally observed from the 2nd to the 4th week of treatment [17,18], but they can also manifest after the conclusion of an acute TMS trial [19]. The delayed response should certainly be considered in the application of this technique, as many patients continue to experience subjective distress and functional impairment until an optimal response is achieved [20]. With those premises, accelerated repetitive TMS (arTMS) represents an innovative form of non-invasive brain stimulation, aimed at enhancing efficacy and shortening response times: arTMS protocols involve multiple daily sessions over consecutive days [21,22]. The rationale for accelerated approaches rests on the assumption that repeated stimulation within a condensed timeframe and tightly scheduled sessions can yield sustained efficacy [23]. One of the first studies to evaluate the efficacy of arTMS was conducted by Holtzheimer and colleagues with an open-label protocol showing a significant clinical effect on depression and anxiety symptoms that was maintained for six weeks after the treatment started [24]. Another single-blind randomized study by Seok and Chung assessed the efficacy and safety of accelerated protocols compared with conventional rTMS in a cohort of twenty MDD patients: the results demonstrated that arTMS was both safe and effective in reducing depressive symptoms over a three-week follow-up period, with no significant differences observed between the two treatment modalities [25]. The same results were observed in a more recent study, showing no significant differences in remission or response rates or reduction in depressive symptoms between the accelerated and standard rTMS treatment groups [26]. Subsequent meta-analyses and systematic reviews have corroborated the promising results regarding the efficacy and non-inferiority of arTMS compared to standard TMS, highlighting its potential to significantly reduce treatment duration and costs [21,22,23].

### 1.3. Aim of the Study

The different parameters and protocols used in studies pose challenges in defining optimal dosing strategies. While most participants showed a good tolerance, ideal parameters such as total stimuli, daily sessions, and inter-session intervals are still difficult to define [20]. In light of the above, this study aimed to assess a sample of patients with TRD in a real-world setting, treated with rTMS and arTMS protocols to evaluate the effectiveness of the two treatment modalities in a three-month follow-up period.

## 2. Materials and Methods

We conducted a cohort study at the ASST Fatebenefratelli-Sacco in Milan, Italy, comparing the clinical outcomes of two groups of patients with a diagnosis of treatment-resistant depressive episodes, within recurrent major depression or bipolar disorder, treated with two different protocols of rTMS in the period between November 2020 and December 2023. Particularly, we compared the clinical outcomes in subjects treated with standard and accelerated protocols of rTMS.

### 2.1. Data Collection and Sample Features

This study included all the consecutive subjects treated with rTMS in our outpatient clinic. Exclusion criteria were the general TMS treatment contraindications. Specifically, exclusion criteria were positive history/family history of epilepsy, serious organic or neurological conditions, substance use disorder (except for nicotine) in the last six months, cognitive impairment, pregnancy, and being in the first six months of post-partum. Informed consent for data collection for research purposes was obtained before starting the treatment. Data were collected anonymously by a trained psychiatrist or a psychiatry resident. The research project complied with the principles of the Declaration of Helsinki regarding medical research in humans, following research ethical requirements. Approval was granted by the Ethics Committee of Milan Area 1, n. 5041, 2020. 

Data regarding sociodemographic features, family psychiatric history, diagnosis, age at the onset, duration of illness, number of lifetime depressive and manic episodes, duration of current episode, psychiatric and other comorbidities, lifetime and current suicidality, and type of pharmacological or psychotherapy treatments were collected.

Psychometric assessment was made using Hamilton Depression Rating Scale 21 items (HAM-D21) [27], Hamilton Anxiety Rating Scale (HAM-A) [28], Montgomery–Asberg Depression Rating Scale (MADRS) [29], Young Mania Rating Scale (YMRS) [30], Self-rating Depression Scale (SDS) [31], Montreal Cognitive Assessment (MoCA) [32], Clinical Global Impression Severity of Illness (CGI-S) [33], and Columbia Suicide Severity Rating Scale (C-SSRS) [34]. Subjects were assessed at four timepoints: before starting treatment (T0), one week after the treatment ended (T1), and one month (T2) and three months (T3) after the treatment ended.

The total sample size was 28 subjects; 9 were treated with the 4-week (standard) protocol, and 19 were treated with the 2-week (accelerated) protocol.

### 2.2. TMS Protocols

Subjects were treated according to the most recent Safety Guidelines for TMS use [35], following two different protocols:

For the rTMS protocol (4-week standard protocol), patients received one session per day from Monday to Friday for four weeks, with the following parameters: stimulation over the left DLPFC, high frequency (10 Hz), at 120% of the motor threshold (MT), and 11 trains of 30 s each, interspersed by 30 s of pause (3000 stimuli per session).

For the arTMS protocol (2-week accelerated protocol), patients received two sessions per day from Monday to Friday for two weeks, with the following parameters: stimulation over the left DLPFC, high frequency (10 Hz), at 120% of the motor threshold (MT), and 11 trains of 30 s each, interspersed by 30 s of pause (3000 stimuli per session).

TMS protocol descriptions are summarized in Table 1.

Previous pharmacological treatments were not discontinued.

### 2.3. Statistical Analysis

Statistical analysis was conducted using RStudio version 4.3.3.

Since we could not assume a normal distribution, continuous variables were described using the median and interquartile range (IQR). The frequency of categorical variables was expressed as both the number and percentage of cases. Continuous variables were compared using the Mann–Whitney U test, while the Chi-square test was employed to compare categorical variables.

One subject for each protocol type dropped out of the study. In more detail, for the standard protocol, one subject dropped out during the first week of treatment, while, for the accelerated protocol, one subject dropped out at the end of the first week for clinical worsening. All the other subjects completed the protocol treatment, but 4 subjects were partially lost at follow-up. For the MoCA test, one subject treated with the standard protocol did not complete the evaluation at any timepoint. Missing data were considered as “Missing at Random” (MAR) and imputed using the predictive mean matching in a model of “Multiple Imputation by Chained Equation” (MICE) [36].

Differences in the scores of HAM-D, HAM-A, MADRS, YMRS, SDS, MoCA, CGI, and C-SSRS in T0, T1, T2, and T3 were evaluated using the Mann–Whitney U test using Benjamini–Hochberg *p*-value adjustment method.

A linear mixed-effect regression (LMER) was used to determine if the observed differences in the scores of HAM-D, HAM-A, MADRS, YMRS, SDS, MoCA, CGI, and C-SSRS followed a trend dependent on the protocol adopted. We built two different models that considered the trend in psychometric evaluation over time accounting for the effect of the two different protocols: the first model was built without considering an interaction between the protocol type and the timepoint, while the second one accounted for that. Then, we compared the two models with a model that did not consider the possible effect of the protocol type, and with a model that considered only a random intercept. Analysis of deviance in covariates was assessed using the Wald test. Comparison between models was made using the Likelihood Ratio Test (LRT).

LMER assumptions of variance homogeneity and normal distribution of the residuals were checked.

A *p*-value of less than 0.05 was deemed to indicate statistical significance.

## 3. Results

The total sample size was 28 subjects; 9 were treated with the 4-week (standard) protocol, and 19 were treated with the 2-week (accelerated) protocol. Considering the whole sample, the median age (years) was 55 [IQR: 42.75, 58.75], and 64.3% were females. No significant differences in sociodemographic and clinical features were found, as shown in Table 2. Considering the whole sample, the number of subjects with bipolar disorder was 8 (28.57%): 1 (11.11%) subject in the standard treatment and 7 (36.84%) in the accelerated one. Analysis of treatment efficacy for the two diagnostic groups was not performed given the small sample size of bipolar patient subgroups.

**Figure 1:**
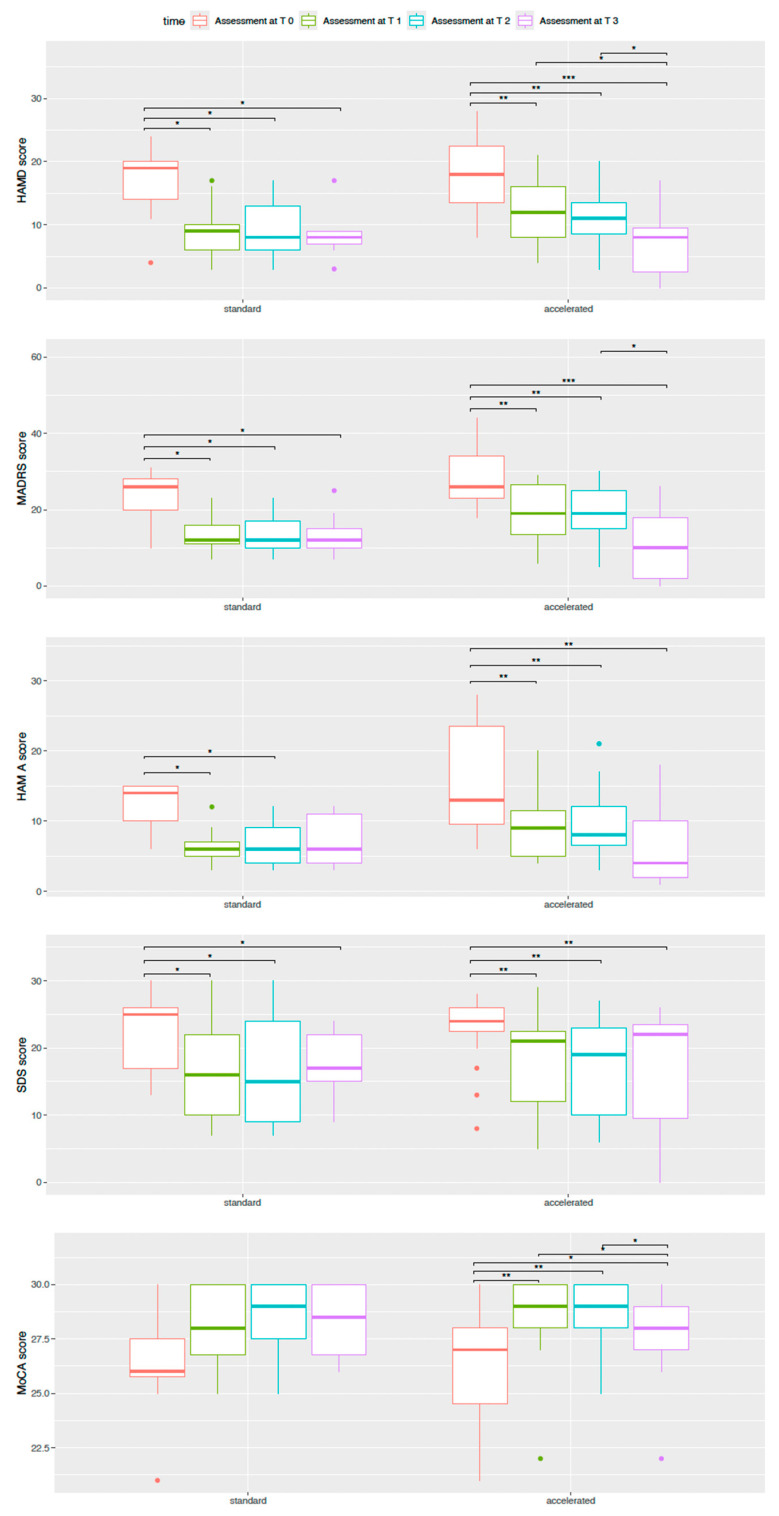
HAMD, MADRS, HAMA, SDS, and MOCA scores over time in the two groups (* *p*-value ≤ 0.05; ** *p*-value ≤ 0.01; and *** *p*-value ≤ 0.01). HAM-D21: Hamilton Depression Rating Scale 21 items; HAM-A: Hamilton Anxiety Rating Scale; MADRS: Montgomery–Asberg Depression Rating Scale; YMRS: Young Mania Rating Scale; SDS: Self-rating Depression Scale; MoCA: Montreal Cognitive Assessment; CGI-S: Clinical Global Impression Severity of Illness; C-SSRS: Columbia Suicide Severity Rating Scale.

Regarding psychometric evaluation at T0, no statistically significant differences were found between the two samples (Table 3). Headaches were reported at the end of the stimulation sessions by four patients. The total sample HAM-D21 score decreased over time (T0: 18.50 [13.75, 21.00], T1: 11.50 [6.75, 15.25], T2: 10.50 [7.75, 13.25], T3: 8.00 [5.25, 9.00]); significant differences were found comparing scores at T0 with T1, T2, and T3 (*p* ≤ 0.001). Differences between T1 and T3 (*p* ≤ 0.05) and T2 and T3 (*p* ≤ 0.05) were also significant. Comparing individuals who received the standard protocol (T0: 19.00 [14.00, 20.00], T1: 9.00 [6.00, 10.00], T2: 8.00 [6.00, 13.00], T3: 8.00 [7.00, 9.00]), a significant decrease was found in the HAM-D21 score at T0 compared with T1, T2, and T3 (*p* ≤ 0.05). Regarding subjects who received the accelerated protocol (T0: 18.00 [13.50, 22.50], T1: 12.00 [8.00, 16.00], T2: 11.00 [8.50, 13.50], T3: 8.00 [2.50, 9.50]), differences found in T0 with T1, T2, and T3 (*p* ≤ 0.01), and differences in T1 compared with T2 and T2 compared with T3 (*p* ≤ 0.05) were significant. Considering the total sample HAM-A score, a significant decrease over time was found (T0: 13.50 [9.75, 16.00], T1: 7.00 [5.00, 10.00], T2: 7.50 [5.75, 11.25], T3: 4.50 [2.00, 11.00]); indeed, differences between T0 and T1, T2, and T3 were significant (*p* ≤ 0.001). The same differences were observed in subjects treated with the accelerated protocol (T0: 13.00 [9.50, 23.50], T1: 9.00 [5.00, 11.50], T2: 8.00 [6.50, 12.00], T3: 4.00 [2.00, 10.00]) (*p* ≤ 0.01). Conversely, considering only subjects who received the standard protocol (T0: 14.00 [10.00, 15.00], T1: 6.00 [5.00, 7.00], T2: 6.00 [4.00, 9.00], T3: 6.00 [4.00, 11.00]), a significant decrease comparing T0, T1, and T2 was found (*p* ≤ 0.01). A significant decrease in MADRS score was observed (T0: 26.00 [23.00, 30.25], T1: 17.50 [11.00, 24.00], T2: 17.50 [13.50, 23.25], T3: 10.50 [2.00, 18.00]); particularly, differences between T0 and T1, T2, and T3 (*p* ≤ 0.001) and between T2 and T3 (*p* ≤ 0.05) were significant. Considering subjects treated with the standard protocol (T0: 26.00 [20.00, 28.00], T1: 12.00 [11.00, 16.00], T2: 12.00 [10.00, 17.00], T3: 12.00 [10.00, 15.00]), statistically significant differences between T0 and T1, T2, and T3 were registered (*p* ≤ 0.05). Conversely, for patients who received the accelerated protocol, significant differences were observed not only between T0 and the following timepoints (*p* ≤ 0.01) but also between T2 and T3 (T0: 26.00 [23.00, 34.00], T1: 19.00 [13.50, 26.50], T2: 19.00 [15.00, 25.00], T3: 10.00 [2.00, 18.00]) (*p* ≤ 0.05). The SDS score decreased over time with significant differences in T1, T2, and T3 when compared to T0 (T0: 24.50 [19.75, 26.00], T1: 19.50 [10.00, 22.25], T2: 18.00 [9.75, 23.00], T3: 19.00 [10.00, 23.00]) (*p* ≤ 0.001); the same differences were observed comparing subjects treated following the standard protocol (T0: 25.00 [17.00, 26.00], T1: 16.00 [10.00, 22.00], T2: 15.00 [9.00, 24.00], T3: 17.00 [15.00, 22.00]) (*p* ≤ 0.05) or the accelerated one (T0: 24.00 [22.50, 26.00], T1: 21.00 [12.00, 22.50], T2: 19.00 [10.00, 23.00], T3: 22.00 [9.50, 23.50]) (*p* ≤ 0.01). Considering the MoCA scores, an increase over time was registered (T0: 27.00 [25.00, 28.00], T1: 29.00 [28.00, 30.00], T2: 28.00 [27.00, 29.50], T3: 29.00 [28.00, 30.00]): significant differences in T1, T2, and T3 if compared to T0 (*p* ≤ 0.01) were registered, while a slight decrease in T3 when compared to T2 (*p* ≤ 0.05) was observed. Comparing only individuals treated with the accelerated protocol (T0: 27.00 [24.50, 28.00], T1: 29.00 [28.00, 30.00], T2: 29.00 [28.00, 30.00], T3: 28.00 [27.00, 29.00]), an increase in MoCA score in T1, T2, and T3 compared to T0 (*p* ≤ 0.05) was registered, despite, in T3, a slight decrease in comparison with T1 and T2 being observed (*p* ≤ 0.05). No significant differences in MoCA scores among individuals treated with the standard protocol were found (T0: 26.00 [25.75, 27.50], T1: 28.00 [26.75, 30.00], T2: 29.00 [27.50, 30.00], T3: 28.50 [26.75, 30.00]). The CGI-S score decreased over time showing significant differences in T1, T2, and T3 compared to T0 (T0: 5.00 [4.00, 5.00], T1: 4.00 [3.75, 4.25], T2: 4.00 [3.00, 4.00], T3: 3.50 [2.75, 4.00]) (*p* ≤ 0.001). The same differences were found in individuals who received the accelerated protocol as a treatment (T0: 5.00 [4.00, 5.00], T1: 4.00 [4.00, 5.00], T2: 4.00 [3.50, 4.00], T3: 3.00 [2.00, 4.00]) (*p* ≤ 0.05), while no significant differences were observed in those treated with the standard one (T0: 5.00 [4.00, 5.00], T1: 4.00 [3.00, 4.00], T2: 4.00 [3.00, 4.00], T3: 4.00 [3.00, 4.00]). The score at C-SSR decreased over time with significant differences in T1, T2, and T3 compared to T0 (T0: 0.50 [0.00, 2.00], T1: 0.00 [0.00, 0.00], T2: 0.00 [0.00, 0.00], T3: 0.00 [0.00, 0.00]) (*p* ≤ 0.05). Considering subjects treated with the accelerated protocol (T0: 1.00 [0.00, 1.50], T1: 0.00 [0.00, 0.00], T2: 0.00 [0.00, 0.00], T3: 0.00 [0.00, 0.00), only the reduction in T3 compared to T0 (*p* ≤ 0.05) was significant, while no significant differences were observed in those treated with the standard treatment (T0: 0.00 [0.00, 2.00], T1: 0.00 [0.00, 0.00], T2: 0.00 [0.00, 0.00], T3: 0.00 [0.00, 0.00). No significant differences in YMRS scores over time were found. Boxplots representing the psychometric score modification over time in the two groups are shown in Figure 1.

**Figure 2:**
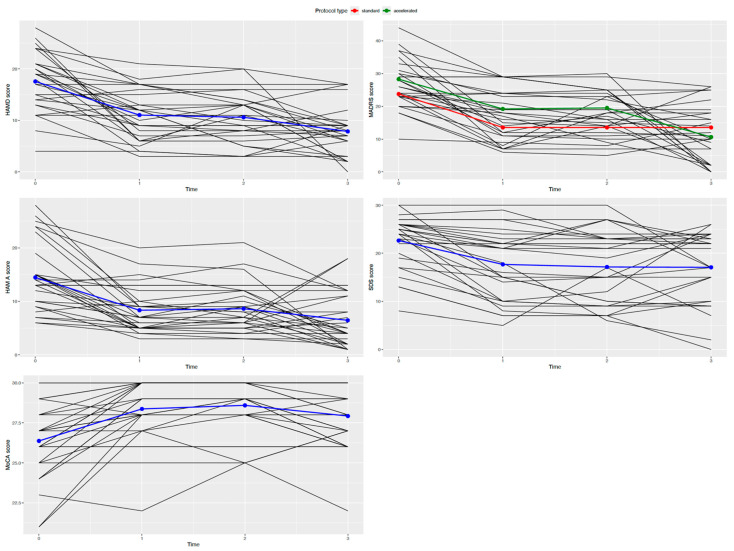
Better fitted models. Legend relates to the MADRS score plot. Blue lines represent HAM-D21, HAM-A, SDS, and MoCA scores over time for the whole sample. HAM-D21: Hamilton Depression Rating Scale 21 items; HAM-A: Hamilton Anxiety Rating Scale; MADRS: Montgomery–Asberg Depression Rating Scale; YMRS: Young Mania Rating Scale; SDS: Self-rating Depression Scale; MoCA: Montreal Cognitive Assessment; CGI-S: Clinical Global Impression Severity of Illness; C-SSRS: Columbia Suicide Severity Rating Scale.

Four LMER models for each psychometric test were built: the first model considered just the random intercept, the second one considered the score variation over time, the third model took into account the effect of the protocol type, and the last one considered the interaction between protocol type and score variation over time. LMER assumptions of variance homogeneity and normal distribution of the residuals were not respected for YMRS, CGI-S, and C-SSRS scores. LMER results for HAM-D21, HAM-A, MADRS, SDS, and MoCA scores are summarized in Table 4. In order to adopt the best model, a comparison between models using LRT for each psychometric evaluation was effected. Considering HAM-D21, differences between model 1 and models 2, 3, and 4 were significant (*p* < 0.001). No significant differences in the LRT were observed for the three models. The same results were observed for the HAM-A models (*p* < 0.001), the SDS models (*p* < 0.001), and the MoCA models (*p* < 0.001). Considering the MADRS, differences between model 1 and models 2, 3, and 4 were significant (*p* < 0.001). Moreover, a statistically significant difference between model 2 and model 4 was observed (*p* < 0.05). No other significant differences in the LRT were registered. Better fitted models were model 2 for HAM-D21, HAM-A, SDS, and MoCA, and model 4 for MADRS. Selected models are shown in Figure 2.

## 4. Discussion

The present study aimed to evaluate rTMS and arTMS protocols’ effectiveness in a sample of TRD patients. Regarding sociodemographic and clinical features, no significant differences in patients who received standard or accelerated protocol were observed at the baseline.

### 4.1. Depressive Symptoms

Considering depressive symptoms, a global improvement over time was observed. Particularly, a significant reduction in HAM-D21, SDS, and MADRS scores across time was found, highlighting—once again—that TMS applied to the left DLPFC is an effective method for depressive symptoms’ improvement in TRD patients. Indeed, it is reported, that stimulation over the left DLPFC helps to normalize the functional connection between this region and the anterior cingulate cortex (ACC), improving depressive symptoms [15,37]. The improvement in depressive symptoms shown in our results is consistent with previous findings [38]. Moreover, the improvement observed in the present study confirms the efficacy of the combined use of rTMS and pharmacological treatment, since patients did not discontinue the previous therapy with antidepressants or mood stabilizers [39]. Considering HAM-D21 reduction over time, a significant decrease was observed for the group treated with arTMS between baseline and T1, T2, and T3, between T1 and T2, and T2 compared to T3, while for patients who received the standard rTMS protocol, a significant reduction was observed only between T0 and the subsequent timepoints. Our findings suggest that, for the standard rTMS treatment, the improvement occurred exclusively at the end of the treatment, remaining stable over time. However, considering the LMER analysis, the HAM-D21 score decreased significantly one week after the treatment, remained stable in T2, and significantly decreased again three months after the treatment ended. No significant differences were observed considering the protocol type as a covariate, highlighting the absence of a superior protocol in reducing depressive symptoms assessed with HAM-D21. These findings are consistent with a previous RCT by Fitzgerald and colleagues [26]. Differences observed between LMER and univariate analysis may be related to the small sample size (especially for the standard rTMS arm), leading to a loss of statistical power. However, considering depressive symptoms’ improvement as a MADRS score reduction, our study showed better efficacy of the accelerated protocol. Considering the LMER models, the two protocols showed similar efficacy in reducing the MADRS score one week after the treatment, but the arTMS seemed to be more effective in reducing the MADRS score three months after the treatment ended. To our knowledge, this is the first article showing this difference between the two protocols, excepting an abstract published in 2023 [40]. MADRS is considered more effective in assessing a modification in the disease presentation if compared to the first Hamilton Depression Rating Scale [29]; indeed, it is largely used to compare treatment efficacy for depression. In light of this, in our sample, MADRS scores may have better identified soft changes in depressive symptoms. In spite of that, the present study did not find any difference between the two protocols in SDS scores over time. Moreover, the only significant reduction in SDS was registered at T1. It is possible that the self-reported symptoms improved in T1 when HAMD-21 and MADRS showed a bigger decrease, while at the subsequent timepoints, the smaller improvement registered by the clinicians reflected SDS stability over time.

### 4.2. Anxiety Symptoms

HAM-A showed a global significant reduction over time. Considering the two protocols, no significant difference was observed in anxiety symptoms’ improvement. Moreover, anxiety symptoms showed a decrease in the first week of follow-up and remained stable in the next timepoints. Considering anxiety symptoms as MDD specifiers, it is possible to assume that the anxiety improvement at the first timepoint and the subsequent stability over time might be related to the larger improvement of depressive symptoms shown in T1. In general, regarding rTMS application for anxiety treatment, mixed findings have been reported. Specifically, two studies conducted on rats examined the impact of rTMS on anxiety-related behavior reporting that rTMS resulted in a significant increase in anxiety-related behaviors [41,42]. Conversely, one study focused on TRD treatment showed a significant reduction in mean HAM-A in both subjects with and without comorbid anxiety disorders, demonstrating a rTMS anxiolytic effect [43]. Our findings are in line with the results observed by Holtzheimer and colleagues which evaluated the improvement in anxiety symptoms through the HAM-A scale in MDD patients, showing no difference between arTMS and rTMS protocols [24]. Furthermore, current evidence suggests that the DLPFC is indeed a target for the treatment of anxiety symptoms, and thus, modifying its connectivity with mesocortical structures—known to be dysfunctional in these patients—could potentially lead to a clinical improvement [44].

### 4.3. Cognitive Symptoms

Improvement in cognition over time was observed. A cognitive assessment was conducted using MoCA at baseline and in the follow-up period. In more detail, an increase in MoCA score was observed in T1, T2, and T3 when compared to the baseline evaluation, but a significant decrease was observed in T3 if compared to T1 and T2. The same results were observed in subjects treated with the accelerated protocol, while no significant difference was noted for the standard one. The difference between the two protocols seemed to be related to the small sample size in the standard rTMS arm. Indeed, considering LMER models, no significant difference was observed in considering the protocol type as a covariate. Moreover, the reduction in the total sample MoCA score at three months remained significant. However, despite the MoCA score reduction observed three months after the treatment ended, a general improvement in cognition over time was observed. This could be explained by the DLPFC’s involvement in neurocognitive functions via its connections with frontosubcortical regions, whose dysfunctions are often implicated in neuropsychiatric disorders [45]. Furthermore, rTMS appears to act independently on both neurocognition and depressive symptoms [46]. Indeed, in the present study, it is possible that the first clinical improvement after rTMS treatment regarded cognitive symptoms, while the concurrent depressive symptoms’ reduction was related primarily to pro-cognitive effects. However, it is plausible that the anti-depressive effect was maintained at least for the three months of follow-up for depression and cognition, while pro-cognitive effects decreased. Cognitive effects of rTMS were evaluated by Fitzgerald and colleagues comparing the two different protocols, demonstrating comparable improvements in both types of treatment even though the final assessment was conducted immediately at the end of the treatment rather than during subsequent follow-up [26]. In addition, sets of cognitive tests are frequently employed in most rTMS studies, and these assessments have consistently shown that rTMS does not negatively impact cognitive function. Indeed, several clinical trials have reported improvements in cognition, most commonly in areas such as attention, working memory, and processing speed, with probable downstream effects leading to enhancements in learning, memory, and aspects of executive functioning [47]. Conversely, although concluding that there might be potential improvement, one systematic review noted that the improvement in cognitive domains has not been confirmed in all the selected studies. Therefore, no conclusive evidence can be drawn about the efficacy of rTMS on cognition [48].

### 4.4. Suicidal Ideation and Gravity of Illness

Regarding suicidal ideation over time, a reduction in C-SSRS scores was observed after the treatment when compared to the baseline. Our findings are consistent with those reported in previous studies which considered rTMS as a promising tool in suicide risk reduction [49,50]. However, a more recent systematic review reported a reduction in suicidal ideation in non-TRD patients, but not in TRD subjects [51]. Our results showed an association between rTMS protocols and suicidal ideation reduction in the first week of follow-up. It has to be noticed, however, that the reduction in suicidal risk in T1 remained stable over time since the sample showed a low suicidal risk from baseline. Our results also showed a reduction in CGI-S over time. This finding highlights the efficacy of the treatment in a reduction in clinical picture severity. However, the reduction was not significant in patients treated with standard rTMS protocol: this result might be due to the small sample size in the 4-week rTMS arms.

### 4.5. Study Limitations

The present study had some limitations. First, the open-label design might reduce the generalizability of our findings. Second, the small sample size—especially in the 4-week standard rTMS arm—could also represent a limitation in reducing statistical power. Finally, the observational design and the absence of randomization could reduce the variability of our sample leading to a decrease in the generalizability of our findings.

## 5. Conclusions

Despite some limitations, this study showed an improvement in both depressive, anxious, and cognitive symptoms in patients treated with rTMS. Considering the two different protocols, the efficacy seemed comparable at one week, while a better clinical response in patients treated with the accelerated protocol was observed in the three-month follow-up. Further studies exploring a longer follow-up period are required to completely understand the long-term efficacy of rTMS. Moreover, a randomized design is needed to better evaluate the differences between the two protocols. Nevertheless, when considering the clinical applications of our findings, it is important to note that, while the improvement in depressive symptoms with the standard protocol is observed after 4 weeks of treatment, the accelerated protocol achieves an equally effective response in a shorter and quicker period, as observed in our study. Reducing the number of treatment days may lead to improved cost-effectiveness, increase the number of patients who can be treated in a given time frame, and enhance accessibility for patients.

## Figures and Tables

**Table 1:** TMS protocol comparisons.

	rTMS (Standard)	arTMS (Accelerated)
N patients	9	19
Duration of the treatment (weeks)	4	2
Sessions per day	1	2
Stimulation site	Left DLPFC	Left DLPFC
Frequency (Hz)	10	10
Intensity	120% of MT	120% of MT
Stimuli per session	3000	3000

rTMS: repetitive transcranial magnetic stimulation; arTMS: accelerated repetitive transcranial magnetic stimulation; DLPFC: dorsolateral prefrontal cortex; MT: motor threshold.

**Table 2:** Sociodemographic and clinical features.

	Total Sample	Standard	Accelerated
*n*	28	9	19
Age (median [IQR], years)	55.00 [42.75, 58.75]	55.00 [49.00, 57.00]	55.00 [41.50, 61.00]
Gender, female (%)	18 (64.29)	7 (77.78)	11 (57.89)
Diagnosis (%)			
Unipolar depression	20 (71.43)	8 (88.89)	12 (63.16)
Bipolar depression	8 (28.57)	1 (11.11)	7 (36.84)
Family history (%)	25 (89.29)	7 (77.78)	18 (94.74)
Marital status (%)			
Single	7 (25.00)	2 (22.22)	5 (26.32)
Engaged/married	18 (64.29)	5 (55.56)	13 (68.42)
Separated/divorced	3 (10.71)	2 (22.22)	1 (5.26)
Employment (%)			
Full-time	12 (42.86)	4 (44.44)	8 (42.11)
Part-time	3 (10.71)	1 (11.11)	2 (10.53)
Unemployed	8 (28.57)	4 (44.44)	4 (21.05)
Retired	3 (10.71)	0 (0)	3 (15.79)
Housewife	2 (7.14)	0 (0)	2 (10.53)
Educational status (%)			
Primary school	1 (3.57)	0 (0)	1 (5.26)
Secondary school	6 (21.43)	3 (33.33)	3 (15.79)
High school	11 (39.29)	2 (22.22)	9 (47.37)
Degree	10 (35.71)	4 (44.44)	6 (31.58)
Age at psychiatric onset (years) (median [IQR])	30.00 [24.75, 45.00]	30.00 [24.00, 36.00]	30.00 [25.50, 45.00]
Duration of illness (months) (median [IQR])	210.00 [93.00, 315.00]	204.00 [132.00, 312.00]	216.00 [78.00, 342.00]
N of lifetime episodes (median [IQR])	4.00 [3.00, 6.25]	3.00 [3.00, 4.00]	5.00 [3.00, 9.00]
N lifetime depressive episodes (median [IQR])	4.00 [2.75, 6.00]	3.00 [2.00, 4.00]	5.00 [3.00, 6.50]
N lifetime hypo/manic episodes	0	0	0
Duration of last episode (days) (median [IQR])	150.00 [60.00, 260.00]	180.00 [60.00, 240.00]	120.00 [75.00, 280.00]
Psychiatric comorbidities (%)	13 (46.43)	4 (44.44)	9 (47.37)
Medical comorbidities (%)	13 (46.43)	4 (44.44)	9 (47.37)
Current psychotherapy (%)	13 (46.43)	5 (55.56)	8 (42.11)
Assuming antidepressants (%)	27 (96.43)	9 (100.00)	18 (94.74)
Assuming mood stabilizers (%)	10 (35.71)	3 (33.33)	7 (36.84)
Assuming antipsychotics (%)	19 (67.86)	7 (77.78)	12 (63.16)
Assuming benzodiazepine (%)	14 (50.00)	6 (66.67)	8 (42.11)
DUI (months) (mean [SD])	10.59 [26.19]	24.44 [42.59]	3.67 [6.55]
Suicidal thoughts lifetime (%)	20 (71.43)	6 (66.67)	14 (73.68)
Suicide attempts lifetime (%)	5 (17.86)	2 (22.22)	3 (15.79)
Current suicidal thoughts (last month) (%)	9 (32.14)	3 (33.33)	6 (31.58)
Current suicide attempts (last 3 months) (%)	2 (7.14)	0 (0)	2 (10.53)

DUI: duration of untreated illness.

**Table 3:** Psychometric evaluation at T0.

	Overall	Standard	Accelerated
*n*	28	9	19
HAM-D21 T0 (median [IQR])	18.50 [13.75, 21.00]	19.00 [14.00, 20.00]	18.00 [13.50, 22.50]
HAM-A T0 (median [IQR])	13.50 [9.75, 16.00]	14.00 [10.00, 15.00]	13.00 [9.50, 23.50]
MADRS T0 (median [IQR])	26.00 [23.00, 30.25]	26.00 [20.00, 28.00]	26.00 [23.00, 34.00]
YMRS T0 (median [IQR])	0.00 [0.00, 0.25]	0.00 [0.00, 0.00]	0.00 [0.00, 0.50]
SDS T0 (median [IQR])	24.50 [19.75, 26.00]	25.00 [17.00, 26.00]	24.00 [22.50, 26.00]
MoCA T0 (median [IQR]) *	27.00 [25.00, 28.00]	26.00 [25.75, 27.50]	27.00 [25.00, 28.00]
CGI-S T0 (median [IQR])	5.00 [4.00, 5.00]	5.00 [4.00, 5.00]	5.00 [4.00, 5.00]
C-SSRS T0 (median [IQR])	0.50 [0.00, 2.00]	0.00 [0.00, 2.00]	1.00 [0.00, 1.50]

* *n* = 27; 8; and 19. HAM-D21: Hamilton Depression Rating Scale 21 items; HAM-A: Hamilton Anxiety Rating Scale; MADRS: Montgomery–Asberg Depression Rating Scale; YMRS: Young Mania Rating Scale; SDS: Self-rating Depression Scale; MoCA: Montreal Cognitive Assessment; CGI-S: Clinical Global Impression Severity of Illness; C-SSRS: Columbia Suicide Severity Rating Scale.

**Table 4:** LMER results.

	Estimate	95% Confidence Interval		*p*-Value
HAM-D21				
Empty model	score ~ 1 + (1|ID)			
(Intercept)	11.768	10.390	13.145	**≤0.001**
2. Treatment effect over time	score ~ D1 + D2 + D3 + (1|ID)			
(Intercept)	17.571	15.690	19.453	**≤0.001**
D1	−6.536	−8.630	−4.442	**≤0.001**
D2	−0.429	−2.523	1.665	0.689
D3	−2.750	−4.844	−0.656	**0.012**
3. Treatment effect over time stratified by protocol (standard as reference)	score ~ D1 + D2 + D3 + protocol + (1|ID)			
(Intercept)	16.581	13.832	19.331	**≤0.001**
D1	−6.536	−8.630	−4.442	**≤0.001**
D2	−0.429	−2.523	1.665	0.689
D3	−2.750	−4.844	−0.656	**0.012**
Protocol type (accelerated)	1.459	−1.494	4.412	0.342
4. Treatment effect over time stratified by protocol (standard as reference), accounting for interaction between protocol and timepoints	score ~ D1 + D2 + D3 + protocol + D1*protocol + D2*protocol + D3*protocol + (1|ID)			
(Intercept)	16.444	13.120	19.769	**≤0.001**
D1	−7.333	−11.035	−3.632	**≤0.001**
D2	0.111	−3.591	3.813	0.953
D3	−0.889	−4.591	2.813	0.639
Protocol type (accelerated)	1.661	−2.375	5.697	0.423
D1*protocol type (accelerated)	1.175	−3.318	5.669	0.610
D2*protocol type (accelerated)	−0.795	−5.289	3.698	0.730
D3*protocol type (accelerated)	−2.743	−7.236	1.751	0.235
HAM-A				
Empty model	score ~ 1 + (1|ID)			
(Intercept)	9.464	8.165	10.764	**≤0.001**
2. Treatment effect over time	score ~ D1 + D2 + D3 + (1|ID)			
(Intercept)	14.464	12.565	16.363	**≤0.001**
D1	−6.143	−8.404	−3.882	**≤0.001**
D2	0.286	−1.975	2.547	0.805
D3	−2.143	−4.404	0.118	0.067
3. Treatment effect over time stratified by protocol (standard as reference)	score ~ D1 + D2 + D3 + protocol + (1|ID)			
(Intercept)	13.028	10.396	15.659	**≤0.001**
D1	−6.143	−8.404	−3.882	**≤0.001**
D2	0.286	−1.975	2.547	0.805
D3	−2.143	−4.404	0.118	0.067
Protocol type (accelerated)	2.117	−0.600	4.834	0.139
4. Treatment effect over time stratified by protocol (standard as reference), accounting for interaction between protocol and timepoints	score ~ D1 + D2 + D3 + protocol + D1*protocol + D2*protocol + D3*protocol + (1|ID)			
(Intercept)	12.333	9.025	15.642	**≤0.001**
D1	−5.889	−9.868	−1.909	**0.005**
D2	0.000	−3.979	3.979	1.000
D3	0.444	−3.535	4.424	0.827
Protocol type (accelerated)	3.140	−0.876	7.157	0.129
D1*protocol type (accelerated)	−0.374	−5.205	4.457	0.880
D2*protocol type (accelerated)	0.421	−4.410	5.252	0.865
D3*protocol type (accelerated)	−3.813	−8.644	1.018	0.126
MADRS				
Empty model	score ~ 1 + (1|ID)			
(Intercept)	18.357	16.529	20.185	**≤0.001**
2. Treatment effect over time	score ~ D1 + D2 + D3 + (1|ID)			
(Intercept)	26.857	24.010	29.704	**≤0.001**
D1	−9.464	−13.028	−5.900	**≤0.001**
D2	0.214	−3.350	3.778	0.906
D3	−6.036	−9.600	−2.472	**0.001**
3. Treatment effect over time stratified by protocol (standard as reference)	score ~ D1 + D2 + D3 + protocol + (1|ID)			
(Intercept)	24.611	20.809	28.413	**≤0.001**
D1	−9.464	−13.028	−5.900	**≤0.001**
D2	0.214	−3.350	3.778	0.906
D3	−6.036	−9.600	−2.472	**0.001**
Protocol type (accelerated)	3.310	−0.470	7.090	0.098
4. Treatment effect over time stratified by protocol (standard as reference), accounting for interaction between protocol and timepoints	score ~ D1 + D2 + D3 + protocol + D1*protocol + D2*protocol + D3*protocol + (1|ID)			
(Intercept)	23.778	18.904	28.652	**≤0.001**
D1	−10.222	−16.346	−4.098	**0.002**
D2	0.000	−6.124	6.124	1.000
D3	0.000	−6.124	6.124	1.000
Protocol type (accelerated)	4.538	−1.379	10.455	0.136
D1*protocol type (accelerated)	1.117	−6.317	8.551	0.769
D2*protocol type (accelerated)	0.316	−7.118	7.750	0.934
D3*protocol type (accelerated)	−8.895	−16.329	−1.461	**0.022**
SDS				
Empty model	score ~ 1 + (1|ID)			
(Intercept)	18.634	16.396	20.872	**≤0.001**
2. Treatment effect over time	score ~ D1 + D2 + D3 + (1|ID)			
(Intercept)	22.643	20.060	25.226	**≤0.001**
D1	−4.964	−7.069	−2.860	**≤0.001**
D2	−0.536	−2.640	1.569	0.619
D3	−0.071	−2.176	2.033	0.947
3. Treatment effect over time stratified by protocol (standard as reference)	score ~ D1 + D2 + D3 + protocol + (1|ID)			
(Intercept)	22.453	18.230	26.677	**≤0.001**
D1	−4.964	−7.069	−2.860	**≤0.001**
D2	−0.536	−2.640	1.569	0.619
D3	−0.071	−2.176	2.033	0.947
Protocol type (accelerated)	0.279	−4.604	5.162	0.912
4. Treatment effect over time stratified by protocol (standard as reference), accounting for interaction between protocol and timepoints	score ~ D1 + D2 + D3 + protocol + D1*protocol + D2*protocol + D3*protocol + (1|ID)			
(Intercept)	22.222	17.584	26.861	**≤0.001**
D1	−5.111	−8.883	−1.339	**0.010**
D2	0.444	−3.328	4.217	0.818
D3	−0.667	−4.439	3.106	0.730
Protocol type (accelerated)	0.620	−5.011	6.251	0.830
D1*protocol type (accelerated)	0.216	−4.363	4.796	0.926
D2*protocol type (accelerated)	−1.444	−6.024	3.135	0.538
D3*protocol type (accelerated)	0.877	−3.702	5.456	0.708
MoCA				
Empty model	score ~ 1 + (1|ID)			
(Intercept)	27.815	27.172	28.458	**≤0.001**
2. Treatment effect over time	score ~ D1 + D2 + D3 + (1|ID)			
(Intercept)	26.370	25.615	27.126	**0.000**
D1	2.000	1.353	2.647	**0.000**
D2	0.222	−0.425	0.869	0.503
D3	−0.667	−1.313	−0.020	**0.047**
3. Treatment effect over time stratified by protocol (standard as reference)	score ~ D1 + D2 + D3 + protocol + (1|ID)			
(Intercept)	26.274	25.007	27.542	**0.000**
D1	2.000	1.353	2.647	**0.000**
D2	0.222	−0.425	0.869	0.503
D3	−0.667	−1.313	−0.020	**0.047**
Protocol type (accelerated)	0.137	−1.299	1.572	0.854
4. Treatment effect over time stratified by protocol (standard as reference), accounting for interaction between protocol and timepoints	score ~ D1 + D2 + D3 + protocol + D1*protocol + D2*protocol + D3*protocol + (1|ID)			
(Intercept)	26.250	24.841	27.659	**0.000**
D1	1.750	0.554	2.946	**0.005**
D2	0.375	−0.821	1.571	0.541
D3	−0.125	−1.321	1.071	0.838
Protocol type (accelerated)	0.171	−1.509	1.851	0.843
D1*protocol type (accelerated)	0.355	−1.070	1.780	0.627
D2*protocol type (accelerated)	−0.217	−1.642	1.208	0.766
D3*protocol type (accelerated)	−0.770	−2.195	0.655	0.293

D1 = 0 in T0, D1 = 1 in T1, T2, and T3; D2 = 0 in T0 and T1, D2 = 1 in T2 and T3; D3 = 0 in T0, T1, and T2, D3 = 1 in T3. The sign “*” is intended as a multiplication sign. HAM-D21: Hamilton Depression Rating Scale 21 items; HAM-A: Hamilton Anxiety Rating Scale; MADRS: Montgomery–Asberg Depression Rating Scale; YMRS: Young Mania Rating Scale; SDS: Self-rating Depression Scale; MoCA: Montreal Cognitive Assessment; CGI-S: Clinical Global Impression Severity of Illness; C-SSRS: Columbia Suicide Severity Rating Scale.

## Data Availability

The data presented in this study are available upon request from the corresponding author due to privacy reasons.
